# Mr. Samuel Knaggs

**Published:** 1911-05-06

**Authors:** 


Mr. Samuel Knaggs, who qualified M.R.C.S., L.S.A. in 1850, has died at Huddersfield, aged eighty-two.

